# Effects of Combined Application of Mushroom Residue and Chemical Fertilizer on Greenhouse Soil Quality and Microbial Community Structure and Function

**DOI:** 10.3390/microorganisms14081605

**Published:** 2026-07-23

**Authors:** Junshen Wang, Kenan Wang, Rui Yuan, Xinyu Xu, Quan Ma, Kaikai Chen, Yingying Jiang, Xiaolong He, Xiangqian Zhang, Xiaodong Liu

**Affiliations:** 1 Shaanxi Key Laboratory of Research and Utilization of Resource Plants on the Loess Plateau, College of Life Sciences, Yan’an University, Yan’an 716000, Chinajyy@yau.edu.cn (Y.J.); ydhelong@163.com (X.H.); zxq@yau.edu.cn (X.Z.); 2 Engineering Research Center of Microbial Resources Development and Green Recycling of Shaanxi Province, Yan’an University, Yan’an 716000, China; 3 Research and Development Centre of Ecological and Sustainable Application of Microbial Industry of the Loess Plateau in Shaanxi Province, Yan’an University, Yan’an 716000, China; 4 Yan’an Junjian Biological Technology Company Limited, Yan’an 716000, China

**Keywords:** protected soil, metagenomic sequencing, soil nutrients, microbial diversity, similarity percentage analysis

## Abstract

To explore the effects of different fertilization regimes on physicochemical properties and microbial ecology of greenhouse soil, we set five treatments with original soil (BS) as the control: chemical fertilizer alone (GF), high/low-rate mushroom residue combined with chemical fertilizer (MH, ML), and high/low-rate organic fertilizer combined with chemical fertilizer (OH, OL). Metagenomic sequencing and bioinformatic analyses were adopted to characterize soil nutrients, microbial communities, and C-N-P-S metabolic functions. All treatments increased soil nutrients. MH had the highest organic matter, total nitrogen, nitrate nitrogen, and available phosphorus, while GF contained the most available potassium and ammonium nitrogen. Bacteria dominated the soil microbiota, with *Pseudomonadota* and *Pseudomonas* as keystone taxa. Mushroom residue amendments improved microbial richness and diversity. By improving soil physicochemical properties, the combined application of organic fertilizer with chemical fertilizer and mushroom residue with chemical fertilizer both enriched some beneficial microorganisms. Chemical fertilizer alone enhanced anaerobic metabolism, which was reversed by high-rate mushroom residue. Available phosphorus, available potassium, and ammonium nitrogen were key environmental factors driving the differentiation of microbial communities and their functions. Overall, mushroom residue combined with chemical fertilizer is effective for greenhouse soil improvement, with proper dosage and tillage recommended.

## 1. Introduction

China had the world’s largest protected cultivation area of about 2.67 million hectares in 2021, accounting for nearly 80% of the global total [1]. As a vital component of modern agriculture in China, protected cultivation has markedly boosted the production efficiency and supply capacity of cash crops, including fruits and vegetables [1]. However, long-term intensive utilization has caused increasingly serious soil quality degradation in protected farmland, which severely restricts the sustainable development of protected agriculture. Soil microorganisms serve as the core drivers of soil nutrient cycling and participate in multiple key ecological processes such as soil organic matter decomposition, nutrient transformation, and pollutant degradation. The stability of microbial community structure and functions can directly reflect soil health status [2].

Soil physicochemical properties, including organic matter, are key environmental factors governing the structure and ecological functions of soil microbial communities. Changes in fertilization regimes can indirectly regulate the composition and functional traits of soil microbes by reshaping the soil’s physicochemical environment [3]. Chemical and organic fertilizers are the most commonly used fertilization practices in protected agriculture. Sole application of chemical fertilizers can rapidly replenish soil available nutrients and boost crop yields in the short term. However, long-term continuous use tends to trigger a series of soil degradation issues, such as soil acidification, nutrient imbalance, and reduced microbial diversity [4,5]. In contrast, organic fertilizer application effectively increases soil organic carbon stock and available nutrient contents, improves soil physicochemical conditions, facilitates the enrichment and proliferation of beneficial microbes, and thereby enhances the stability of soil ecosystems [6]. Current studies worldwide on microbial responses to fertilization mainly focus on the variation patterns and mechanisms of microbial communities under long-term single fertilization. In practical field production, dynamic fertilization regimes such as combined and alternate application of chemical and organic fertilizers are widely adopted to balance nutrient supply efficiency and soil conservation [5,7,8]. Nevertheless, the response mechanisms of soil microbes under these regimes remain to be systematically explored.

China ranks as the world’s largest producer of edible fungi, contributing to over 70% of the global output [9]. A large quantity of mushroom residue waste is generated during cultivation, yet its overall resource utilization rate remains low at approximately 33% [9]. Fresh uncomposted mushroom residue is rich in organic matter and mineral nutrients, and also contains abundant microorganisms and bioactive substances such as enzymes [10,11]. It exerts positive effects on improving soil physicochemical properties and restoring soil microecosystems. Accordingly, mushroom residue can be used as a high-quality organic fertilizer for soil improvement in protected agriculture. Numerous studies have demonstrated that the appropriate application of mushroom residue effectively ameliorates soil properties, improves soil fertility and microbial diversity, and further enhances the yield and quality of greenhouse fruits and vegetables [12,13]. Pintarič et al. reported that mushroom residue application significantly increased the abundance of actinomycetes, fungi, and total microorganisms, as well as the activities of enzymes involved in carbon, nitrogen, phosphorus, and sulfur cycles in various soils [14]. Combined use with conventional fertilizers could produce synergistic effects, indicating that mushroom residue is an excellent organic soil amendment. He et al. also found that moderate addition of mushroom residue markedly raised the contents of soil organic carbon, total nitrogen, and total phosphorus [15]. Meanwhile, it enriched beneficial microorganisms, including *Bacillus*, *Bacteroides*, and *Pseudomonas*, and optimized the structure of soil microbial communities.

To date, many studies have investigated the effects of the application of mushroom residue and organic manure, as well as the combined applications with chemical fertilizers, on soil physicochemical properties and microbial community structure. Nevertheless, limited studies have focused on different fertilization regimes on greenhouse agricultural soils in the Loess Plateau. Soils in this region are generally impoverished, with low organic matter content. To achieve high crop yields, local greenhouse cultivation has long relied on excessive chemical fertilizers, resulting in severe soil degradation and further altering the structural and functional characteristics of soil microbial communities. To restore degraded soil ecosystems, this study took greenhouse agricultural soils in the Loess Plateau as the research object and adopted metagenomic sequencing technology to systematically analyze the changes in physicochemical properties, microbial community structure, and functions of greenhouse soils under the combined application of chemical fertilizers with mushroom residue and organic manure, respectively. The research results aim to provide a theoretical basis and practical guidance for soil improvement and the efficient resource utilization of mushroom residue in greenhouse farmland of the Loess Plateau, and promote the coordinated green and sustainable development of greenhouse agriculture and edible fungus industries.

## 2. Materials and Methods

### 2.1. Study Site Description

The experimental site is located in Hezhuangping Town, Yan’an City, Shaanxi Province, China (109°25′30″ E, 36°41′23″ N). This area is part of the Loess Plateau, and loessial soil is the dominant soil type here. Five fertilization treatments were designed in this experiment: conventional chemical fertilizer (GF), chemical fertilizer combined with high-rate mushroom residue (MH), chemical fertilizer combined with low-rate mushroom residue (ML), chemical fertilizer combined with high-rate organic manure (OH), and chemical fertilizer combined with low-rate organic manure (OL). Each treatment was set up with three biological replicates, yielding a total of 15 experimental plots. The area of each plot was 8 m^2^, and the total area of the experimental field reached 120 m^2^. There is a 20 cm gap between each plot. Before the experiment commenced, topsoil samples were randomly collected from the 0–20 cm soil layer across the field via the multi-point composite sampling method. These samples were defined as original soil (BS) and used for the initial background analysis of the experiment.

The mushroom residue used in this experiment was the cultivation waste of *Pholiota nameko*, which was provided by Yan’an Junjian Biotechnology Co., Ltd. (Yan’an, China). The residue was crushed and then applied directly to the soil. Its basic physicochemical properties are shown as follows: pH 8.77 ± 0.02, organic matter 279.98 ± 39.98 g/kg, cellulose 15.51 ± 0.66%, lignin 17.51 ± 0.47%, hemicellulose 5.41 ± 0.59%, total nitrogen 6.84 ± 0.17 g/kg, total phosphorus 2.70 ± 0.12 g/kg, and total potassium 8.23 ± 0.05 g/kg. The commercial organic fertilizer for the trial was purchased from Shaanxi FuzhouShenlu Ecological Industry Co., Ltd. (Yan’an, China). The chemical fertilizer was formulated with urea (nitrogen fertilizer), calcium superphosphate (phosphorus fertilizer), and potassium sulfate (potassium fertilizer). Detailed fertilization rates for each treatment are presented in Table 1.

### 2.2. Sample Collection

After collecting the original soil samples, fertilization was implemented in accordance with the treatments listed in Table 1. Two months later, soil samples were taken from the 0–20 cm soil layer of each plot and sealed in sterile sampling bags. All samples were delivered to the laboratory for processing on the same day. The soil in each bag was fully homogenized and sieved through a 2 mm mesh. The sieved soil was divided into three subsamples. The first subsample was used for the determination of soil physicochemical properties. The second one was stored in a −80 °C refrigerator for metagenomic sequencing, and the third was retained as backup.

### 2.3. Analysis of Soil Properties

Soil physicochemical indices were determined in accordance with the Methods of Soil Agrochemical Analysis [16]. Soil pH was measured by the potentiometric method at a soil–water ratio of 2.5:1 using a pH meter (PB-10, Sartorius, Goettingen, Germany). Soil organic matter (OM) was determined using the potassium dichromate oxidation-external heating method, and the titration process was performed with a digital bottle-top burette (Titrette, BRAND GMBH + CO KG, Wertheim, Germany). Soil total nitrogen (TN) was analyzed via the Kjeldahl digestion method. Soil alkaline hydrolysis nitrogen (AN) was measured by the alkaline hydrolysis diffusion method. Soil ammonium nitrogen (NH_4_^+^) and nitrate nitrogen (NO_3_^−^) were extracted with potassium chloride. The TN, AN, NH_4_^+^, and NO_3_^−^ were determined using an automatic continuous flow analyzer (Auto Analyzer 3, AA3, SEAL Analytical GmbH, Norderstedt, Germany). Available phosphorus (AP) was extracted with sodium bicarbonate and quantified by the molybdenum-antimony anti-colorimetric method with an ultraviolet-visible spectrophotometer (UV-1900i, Shimadzu Instruments (Suzhou) Co., Ltd., Suzhou, China). Available potassium (AK) was extracted with ammonium acetate and then detected using a flame spectrophotometer (FP6410, INESA Analytical Instrument Co., Ltd., Shanghai, China).

### 2.4. Soil DNA Extraction and Metagenomic Sequencing

DNA was extracted from soil samples using the CTAB method. DNA degradation degree, potential contamination, and DNA concentration were measured using Agilent 5400 (Agilent, Santa Clara, CA, USA). The sequencing library was generated using the NEBNext^®^ Ultra™ DNA Library Prep Kit for Illumina (NEB, Ipswich, MA, USA) following the manufacturer’s recommendations, and index codes were added to each sample. Briefly, the genomic DNA sample was fragmented by sonication to a size of 350 bp. Then, the DNA fragments were end-polished, A-tailed, and ligated with the full-length adapter for Illumina sequencing, followed by further PCR amplification. After PCR products were purified by the AMPure XP system (Beverly, Sherman Oaks, CA, USA). Subsequently, library quality was assessed on the Agilent 5400 system (Agilent, USA) and quantified by QPCR (1.5 nM). The qualified libraries were pooled and sequenced on Illumina platforms with a PE150 strategy, according to the effective library concentration and the amount required.

Raw sequencing data were preprocessed using Kneaddata to remove low-quality reads. Taxonomic annotation and classification of all effective sequences across samples were performed with Kraken2 (confidence: 0.2), and Bracken (default parameters) was subsequently applied to estimate the actual relative abundance of species in each sample [17,18]. Using the HUMAnN (version 2.0) software, quality-controlled sequences were aligned against the UniRef90 protein database via the DIAMOND algorithm. Based on the correspondence between UniRef90 IDs and various functional databases, the annotation information and relative abundance tables of functional databases were generated. The raw sequence data reported in this paper have been deposited in the Genome Sequence Archive [19] in the National Genomics Data Center [20], China National Center for Bioinformation/Belling Institute of Genomics, Chinese Academy of Sciences (GSA: CRA CRA042386), which are publicly accessible at https://ngdc.cncb.ac.cn/gsa, accessed on 7 May 2026.

### 2.5. Statistical Analysis

The experimental results are expressed as the mean ± standard deviation (SD). One-way analysis of variance (ANOVA) was performed for data analysis, and multiple comparisons among treatments were conducted using the LSD test at *p* < 0.05. Redundancy analysis (RDA) was conducted to evaluate the relationships between bacterial communities and environmental factors. Similarity percentage analysis (SIMPER) was used to screen factors that contributed most to the differences across groups. Non-metric multidimensional scaling (NMDS) and principal coordinates analysis (PCoA) were performed based on the Bray–Curtis distance. The Mantel test was applied to examine the correlations and corresponding significance levels between microbial groups, metabolic pathways, and soil physicochemical properties. R software (4.5.2) and RStudio (2025.09.0) were used for data processing and drawing. Adobe Illustrator 2023 (http://www.adobe.com/) was used for format adjustment of figures.

## 3. Results and Discussion

### 3.1. Soil Physicochemical Parameters

After two months of fertilization under different regimes, marked changes were observed in the physicochemical properties of greenhouse soils (Table 2). Given the high organic matter (OM) content in mushroom residue and organic manure [10], soil OM increased significantly after their application, and the OH treatment showed significantly higher values than other treatments (*p* < 0.05). Compared with the original soil (BS), fertilization remarkably raised soil nitrogen, phosphorus, and potassium concentrations. Although organic manure and mushroom residue contain a certain amount of potassium, available potassium (AK) was significantly higher in the GF treatment than in other groups (*p* < 0.05), followed by MH and ML. This phenomenon may be caused by the short experimental period that restricts potassium release from organic materials. Alternatively, humus and microorganisms from organic amendments may adsorb and assimilate partial potassium ions, leading to lower AK in organic-supplemented soils than in the chemical-fertilizer-only group. Available phosphorus (AP) was significantly higher in MH and ML treatments, indicating that mushroom residue application effectively increases soil AP, which is consistent with the findings of Tang et al. [12]. Previous studies have shown that partial substitution of chemical fertilizer with organic manure increases topsoil total nitrogen (TN), enhances plant nitrogen uptake, and reduces soil nitrate-nitrogen (${\mathrm{NO}}_{3}^{-}$-N) accumulation [21]. Since no crops were planted for nitrogen absorption in this experiment, TN, alkaline hydrolysis nitrogen (AN), and ${\mathrm{NO}}_{3}^{-}$-N all increased significantly with mushroom residue and organic manure addition. Urea can be transformed into ammonium-nitrogen (${NH}_{4}^{+}$-N) by soil urease [22], so GF treatment significantly elevated soil NH_4_^+^ content. By contrast, NH_4_^+^ decreased obviously in co-application treatments, which was presumably because mushroom residue and organic manure improved soil microbial nitrification activity and accelerated the conversion of ${NH}_{4}^{+}$-N to nitrite-nitrogen and ${\mathrm{NO}}_{3}^{-}$-N [23]. Soil pH is closely associated with soil nutrients. Yu et al. reported that organic manure application increases soil alkalinity [24]. In this study, soil pH slightly increased under organic manure treatments but showed no significant difference from the original soil (Table 2). Long-term chemical fertilizer application easily causes soil acidification [4], while pH rose slightly in the GF group in this trial, probably owing to the short fertilization period and low fertilizer dosage. High-rate mushroom residue addition markedly increased soil pH, implying a positive correlation between mushroom residue application rate and soil pH. In summary, combined application of mushroom residue and chemical fertilizer effectively improves soil organic matter, nitrogen, and phosphorus contents and optimizes soil nutrient composition; however, excessive mushroom residue input may trigger soil alkalization.

### 3.2. Soil Microbial Community Structure

**Figure 1:**
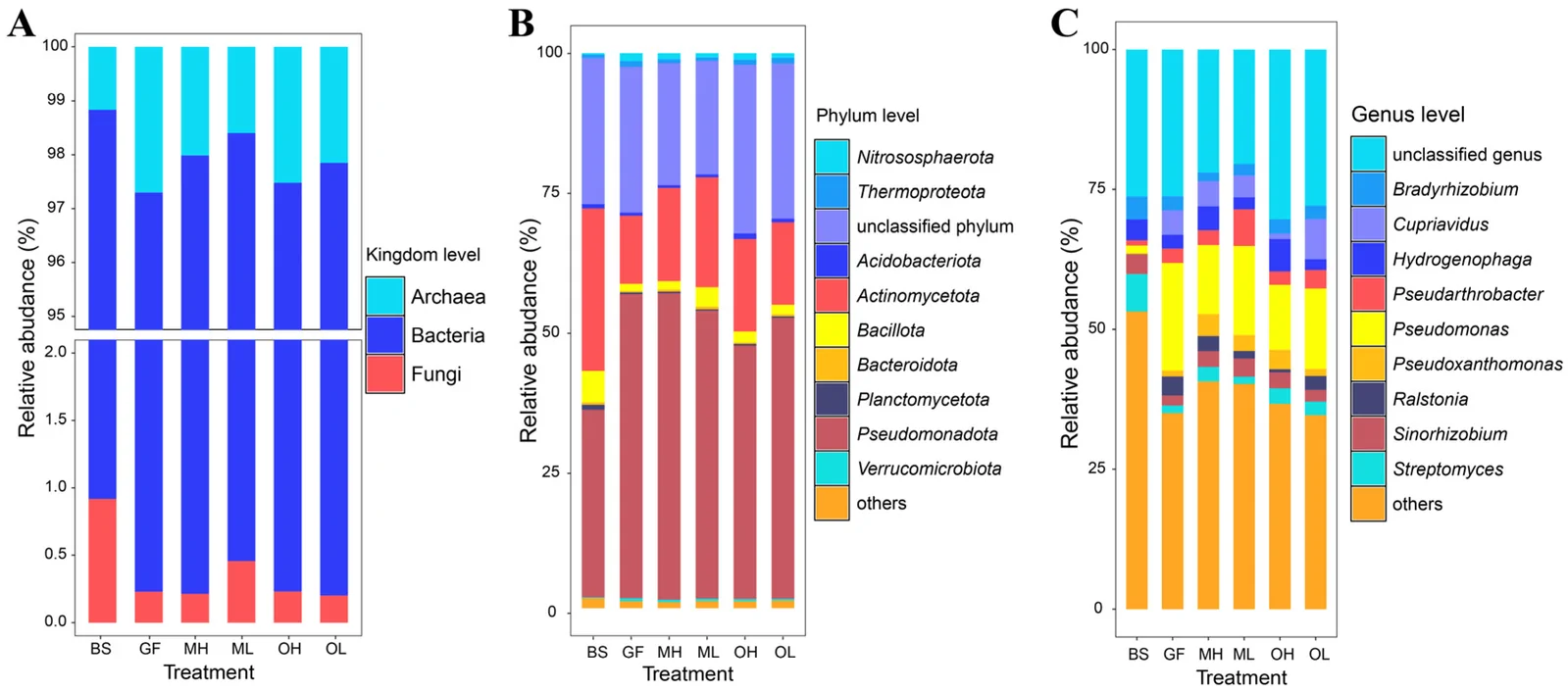
Microbial community structure under different fertilization treatments. (**A**) At kingdom level; (**B**) at phylum level; (**C**) at genus level.

Soil microorganisms are core components of soil ecosystems. They extensively participate in material cycling and energy flow, and play critical roles in maintaining soil ecosystem stability [25,26]. In this study, metagenomic sequencing was used to systematically characterize microbial community structures in the original soil (BS) and soils under five different fertilization treatments, with detailed community composition presented in Figure 1. Kingdom-level analysis showed that Bacteria overwhelmingly dominated all treatments, with relative abundances above 97%. The ML treatment had the highest bacterial abundance of 97.94 ± 0.12%, while GF had the lowest (97.07 ± 0.24%). Archaea was the second-most abundant group, with relative abundances ranging from 1.60 ± 0.10% (ML) to 2.70 ± 0.33% (GF). Fungi exhibited the lowest abundance, varying from 0.20 ± 0.01% (OL) to 0.92 ± 0.22% (BS) (Figure 1A). At the phylum level (Figure 1B), the relative abundance of *Pseudomonadota* significantly increased in all fertilization treatments compared with BS (*p* < 0.05). MH had the highest *Pseudomonadota* abundance (55.27 ± 0.86%), whereas OH had the lowest (45.69 ± 1.68%). Previous studies have confirmed that *Pseudomonadota* abundance is negatively correlated with fungal abundance in eutrophic environments. High-abundance *Pseudomonadota* occupy the ecological niches of fungi, which largely accounts for the generally low fungal abundance in this study [27] (Figure 1A). Opposite to the variation trend of *Pseudomonadota*, *Actinomycetota* showed the highest relative abundance in BS (29.28 ± 5.08%). Except for ML, all other fertilization treatments significantly reduced the abundance of *Actinomycetota* (*p* < 0.05), with GF having the lowest value (12.25 ± 1.12%). The relative abundance of *Bacillota* in all fertilized soils was significantly lower than that in BS (*p* < 0.05). Among the five fertilization groups, GF had the lowest *Bacillota* abundance (1.29 ± 0.23%), and ML the highest (3.52 ± 0.17%). *Nitrososphaerota* (Archaea) is characterized by chemolithoautotrophic metabolism and can oxidize ammonium-nitrogen into nitrite-nitrogen [28]. Compared with BS, all fertilization treatments significantly increased the relative abundance of *Nitrososphaerota* (*p* < 0.05), with no significant differences observed among the five fertilization groups (*p* > 0.05). Soil urea is hydrolyzed into ammonium-nitrogen, providing sufficient nitrogen sources for *Nitrososphaerota* growth, while exogenous organic matter supplements essential carbon sources. Therefore, organic manure treatments (OH, OL) and mushroom residue treatments (MH, ML) effectively enhanced *Nitrososphaerota* abundance, consistent with the findings of Wang et al. [29]. In addition to the above dominant phyla, 20.47–30.39% of microorganisms in all treatments were classified as unassigned phyla. This indicates high diversity of soil microbial resources, with numerous undiscovered microbial taxa awaiting further exploration [30].

At the genus level, fertilization markedly changed the community structure of dominant soil microbes, among which *Pseudomonas* became the absolutely dominant genus in fertilized soils. The relative abundance of *Pseudomonas* was only 1.38 ± 0.35% in the original BS soil, whereas it increased sharply under all fertilization treatments, ranging from 11.63 ± 0.90% in the OH treatment to 19.27 ± 0.92% in the GF treatment. As a core plant-growth-promoting rhizobacteria (PGPR), *Pseudomonas* plays vital roles in soil element cycling and plant growth regulation [31,32]. *Cupriavidus* exhibited a similar variation trend to *Pseudomonas*, with significantly elevated relative abundance after fertilization and the highest value of 7.22 ± 1.65% in the OL treatment. Systematic research on *Cupriavidus* remains limited, and existing studies mainly focus on the plant-growth-promoting functions and heavy-metal resistance mechanisms of the model strain *Cupriavidus metallidurans* [33]. In addition, *Pseudarthrobacter*, *Pseudoxanthomonas,* and *Ralstonia* shared the same abundance pattern as *Pseudomonas*, and their relative abundances were significantly enhanced by all fertilization regimes. *Streptomyces* had the highest relative abundance (6.68 ± 1.75%) in BS, with no significant differences compared with the five fertilization treatments, while its abundance in OH and MH was significantly higher than that in GF. *Streptomyces* is a major producer of novel secondary metabolites and valuable soil biocontrol agents [34,35]. Organic manure and mushroom residue application effectively enriched the soil with bacteria of the genus *Streptomyces*, implying potential inhibitory effects against soil-borne diseases. *Sinorhizobium* showed the highest relative abundance (3.5 ± 0.27%) in BS, which was significantly higher than that in GF (*p* < 0.05), but had no significant differences with organic manure and mushroom residue treatments (*p* > 0.05). As a typical PGPR, *Sinorhizobium* not only achieves symbiotic nitrogen fixation with legumes to improve soil nitrogen nutrition, but some strains also regulate plant growth by secreting active substances such as IAA, contributing greatly to soil amelioration and plant growth promotion [36,37].

In summary, the overall composition of dominant soil microbial communities remained consistent with the original soil after fertilization, while the relative abundances of dominant genera were significantly altered, which was closely related to fertilization regimes. Sole chemical fertilizer application reduced the abundance of beneficial microbes, including *Pseudomonas*, *Sinorhizobium*, and *Streptomyces*. In contrast, combined application with organic manure or mushroom residue could partially restore the population of beneficial microbes and optimize soil microbial community structure.

### 3.3. Microbial Diversity

**Figure 2:**
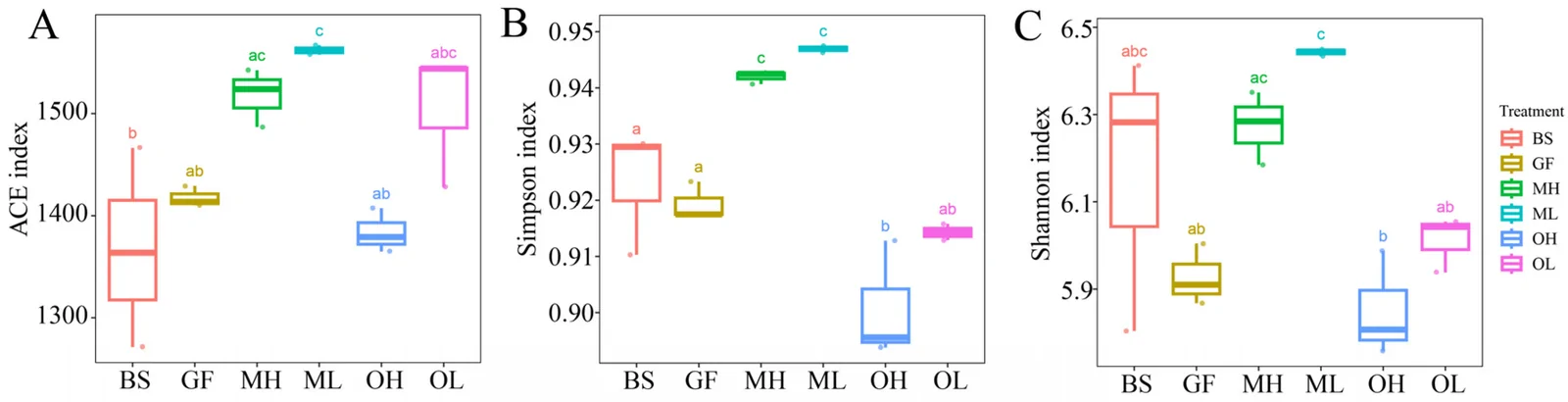
Alpha diversity of soil microbial communities under different fertilization treatments. (**A**) ACE index; (**B**) Simpson index; (**C**) Shannon index. Different lowercase letters indicate significant differences (*p* < 0.05) based on a one-way ANOVA, followed by an LSD test.

The α-diversity of soil microorganisms in the original soil and five fertilization treatments is illustrated in Figure 2. Analysis of soil microbial richness (ACE index) revealed that the ML treatment had a significantly higher ACE index than BS, GF, and OH (*p* < 0.05), with no significant difference from MH and OL. The MH treatment showed a significantly higher ACE index than BS, while no significant differences were found compared with GF, OH, and OL. Although the OL treatment had a relatively high overall ACE index, large intra-group data dispersion led to no significant differences from low-richness treatments such as BS, GF, and OH (Figure 2A). These results indicated that the combined application of mushroom residue and chemical fertilizer effectively improved soil microbial richness. Moreover, treatments with low-rate mushroom residue or organic manure outperformed those with high-rate amendments, which was consistent with previous research [38]. For soil microbial diversity indices, mushroom residue treatments (MH, ML) had significantly higher Simpson indices than all other groups (*p* < 0.05). BS and GF exhibited significantly higher Simpson indices than OH, with no significant difference from OL (Figure 2B). The Shannon index followed a similar pattern to the Simpson index. ML had a significantly higher Shannon index than GF, OH, and OL (*p* < 0.05), and no significant difference with MH and BS. MH had a significantly higher Shannon index than OH (*p* < 0.05), but no difference from ML, BS, GF, and OL. OH had the lowest Shannon index among all treatments, significantly lower than MH and ML, yet comparable with BS, GF, and OL (Figure 2C). Overall α-diversity results demonstrated that short-term single chemical fertilizer application and organic manure–chemical fertilizer co-application exerted limited regulatory effects on soil microbial richness and diversity. In contrast, mushroom residue addition significantly increased both metrics, consistent with the findings of Geng et al. [39]. This could be attributed to the abundant complex nutrients in the mushroom residue [10,11], which provide sufficient substrates for bacterial proliferation and enrichment, thus effectively enhancing soil microbial community diversity.

**Figure 3:**
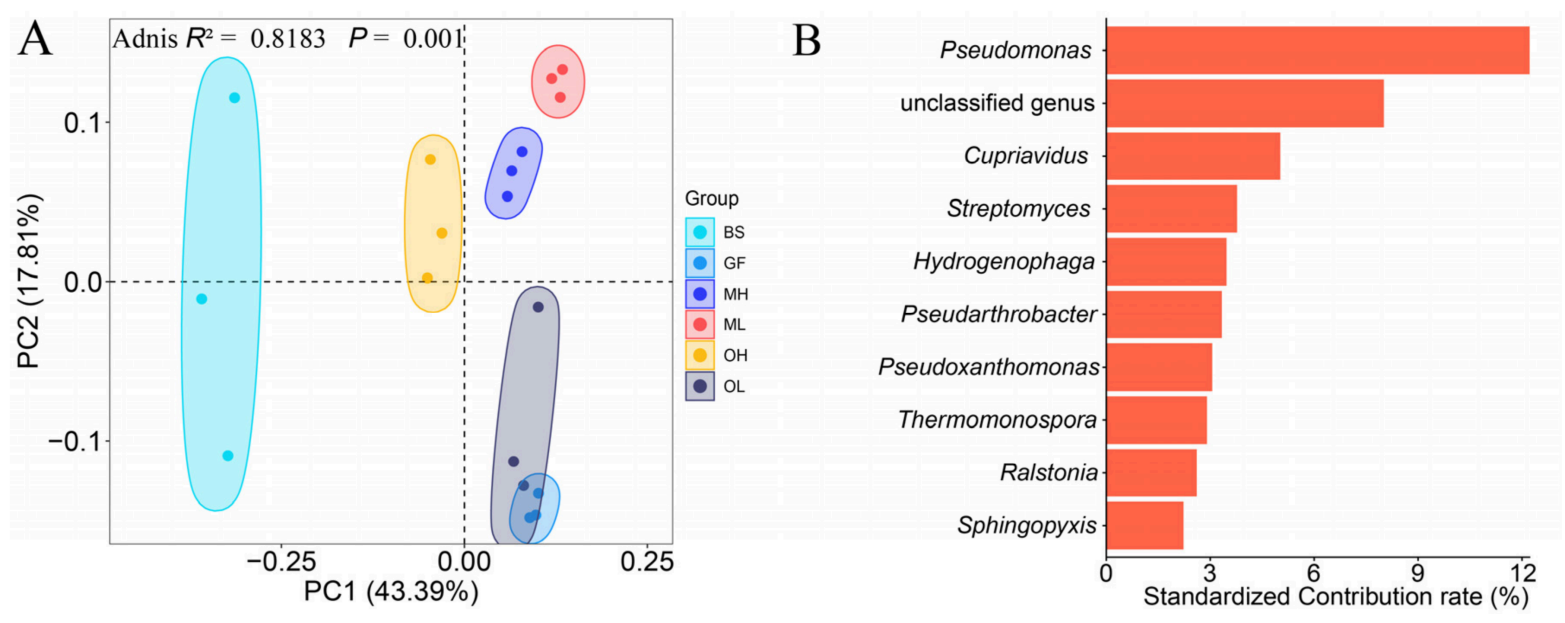
Soil microbial community structure under different fertilization treatments. (**A**) PCoA analysis; (**B**) Simper analysis.

To reveal the differentiation characteristics of soil microbial community structure under different fertilization treatments, principal coordinate analysis (PCoA) based on the Bray–Curtis distance was conducted in this study. Permutational multivariate analysis of variance (Adonis) was adopted to test the significance of community structural differences among treatments, and similarity percentage (SIMPER) analysis was used to identify key microbial taxa driving community divergence (Figure 3). The first principal component (PC1) and the second principal component (PC2) explained 43.39% and 17.81% of the variation in soil microbial community structure, respectively, with a cumulative explanation rate of 61.20%, which could effectively characterize the overall community differences among samples. Adonis analysis verified that microbial community structures differed significantly across treatments, and inter-group differences were markedly larger than intra-group differences (*R*^2^ = 0.8183, *p* = 0.001). SIMPER analysis further screened the top 10 microbial genera contributing to community structural variation: *Pseudomonas*, unclassified genus, *Cupriavidus*, *Streptomyces*, *Hydrogenophaga*, *Pseudarthrobacter*, *Pseudoxanthomonas*, *Thermomonospora*, *Ralstonia*, and *Sphingopyxis*. Among these genera, *Pseudomonas* exhibited the highest standardized contribution rate, followed by unclassified genera, indicating that these two genera were core taxa driving microbial community differentiation.

### 3.4. Effects of Different Fertilization Methods on C, N, P, and S Metabolism

**Figure 4:**
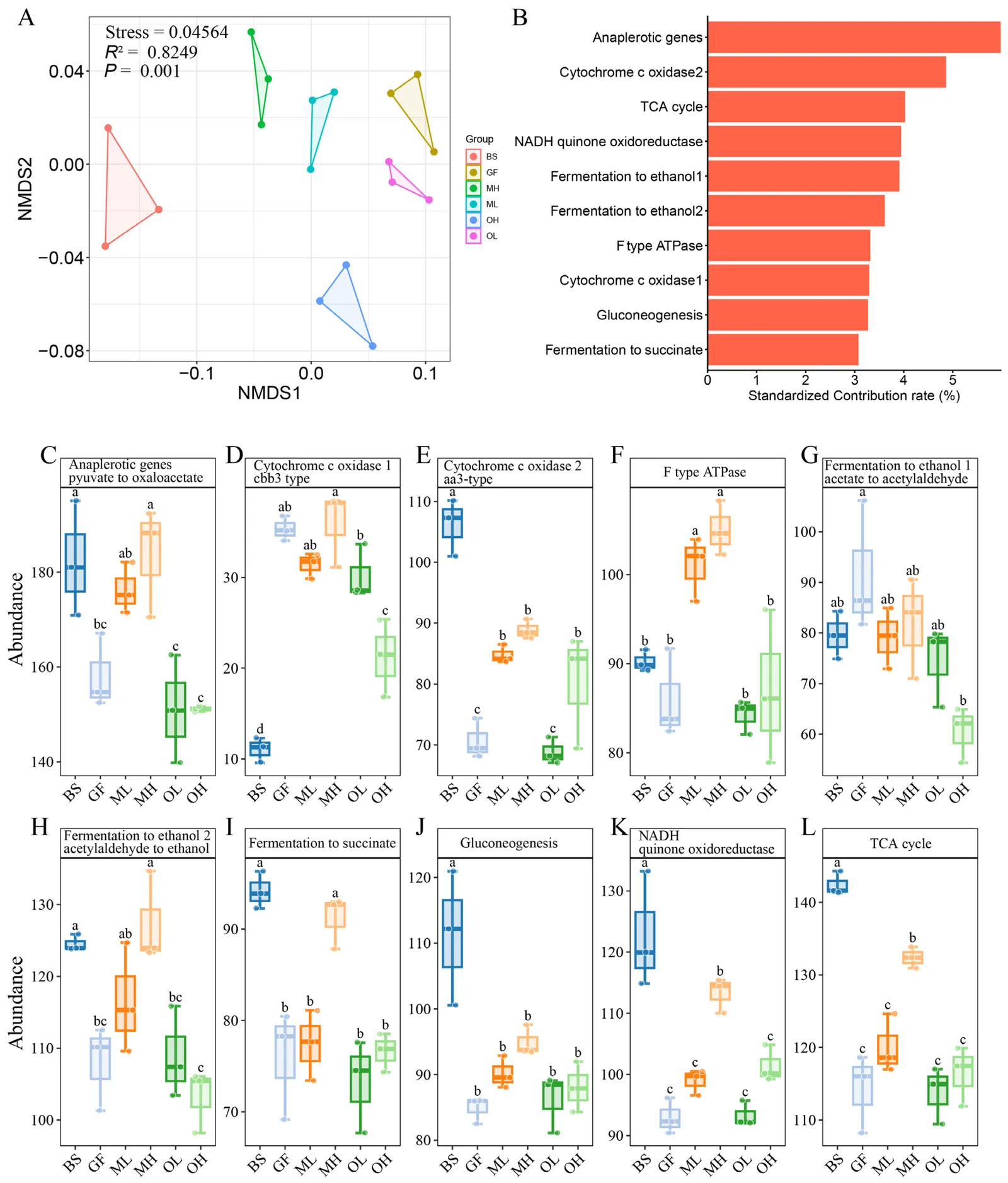
Analysis of differences in carbon, nitrogen, phosphorus, and sulfur metabolic functional genes and gene abundance of key metabolic pathways under different fertilization treatments. (**A**) Non-metric multidimensional scaling analysis based on the Bray–Curtis distance of functional gene abundances related to carbon, nitrogen, phosphorus, and sulfur (C-N-P-S) metabolism; (**B**) Simper analysis based on the functional gene abundances of metabolic pathways related to C-N-P-S metabolism; (**C**–**L**) the total abundance of genes related to each metabolic pathway in different fertilization treatments. Different lowercase letters in (**C**–**L**) indicate significant differences (*p* < 0.05) based on a one-way ANOVA, followed by an LSD test.

To explore the response characteristics of soil microbial carbon, nitrogen, phosphorus, and sulfur metabolic functions under different fertilization treatments, non-metric multidimensional scaling (NMDS) was performed based on the abundance matrix of functional genes related to C-N-P-S metabolism to analyze differences in microbial functional structures among treatments (Figure 4A). Similarity percentage (SIMPER) analysis was adopted to identify key metabolic pathways driving microbial functional differentiation (Figure 4B), and inter-group differences in gene abundance of the top-ten pathways with the highest standardized contribution rates were further compared (Figure 4C–L). NMDS ordination revealed obvious differentiation in the functional structures of soil microbial C-N-P-S metabolism across fertilization regimes. The Adonis test further verified that fertilization patterns extremely significantly altered the C-N-P-S functional structures of soil microbial communities. Inter-group differences were larger than intra-group differences, explaining 82.5% of the variation in microbial functional structures (Stress = 0.046, *R^2^* = 0.825, *p* = 0.001). Hence, fertilization acted as a key factor driving functional divergence of microbial communities (Figure 4A). SIMPER analysis indicated that the top ten key metabolic pathways contributing to functional structural differences all belonged to the carbon cycle. Anaplerotic genes showed the highest contribution rate and served as the core pathway for functional differentiation. The remaining key pathways in sequence were Cytochrome c oxidase 2, tricarboxylic acid (TCA) cycle, NADH quinone oxidoreductase, Fermentation to ethanol 1 and 2, F-type ATPase, Cytochrome c oxidase 1, gluconeogenesis, and Fermentation to succinate. Fertilization significantly modulates soil nutrient contents [39], thereby affecting variations in nutrient-associated metabolic pathways. In this study, the core pathways with high differential contribution were all involved in carbon cycling. This result was closely related to the exogenous input of abundant organic matter via organic manure and mushroom residue. The decomposition and utilization of organic matter substantially reshaped carbon metabolic activities in soils under different treatments, ultimately resulting in significant inter-group differences in carbon metabolic functions [40].

Analysis of total gene abundances of the top-ten key metabolic pathways revealed significant divergence in soil microbial carbon-metabolic patterns among different fertilization treatments. The original BS soil had significantly higher gene abundances related to Cytochrome c oxidase 2 (aa3-type) (Figure 4E), TCA cycle (Figure 4L), gluconeogenesis (Figure 4J), and NADH quinone oxidoreductase (Figure 4K) than all fertilized soils (*p* < 0.05), while it exhibited the lowest abundance of Cytochrome c oxidase 1 (cbb3-type) genes (Figure 4D, *p* < 0.05). Significant differences among GF, ML, and OL were only found in anaplerotic genes (Figure 4C), Cytochrome c oxidase 2 (aa3-type) (Figure 4E), and F-type ATPase (Figure 4F) (*p* < 0.05). No inter-group differences were detected for the other seven pathways (*p* > 0.05), indicating similar overall C-N-P-S metabolic functions across these three treatments. Among the five fertilization regimes, MH maintained relatively high overall gene abundances of metabolic pathways. Genes associated with succinate fermentation (Figure 4I), NADH quinone oxidoreductase (Figure 4K), and TCA cycle (Figure 4L) were significantly more abundant in MH than in other treatments (*p* < 0.05). MH and ML showed no significant differences in these three pathways; however, MH had significantly higher total gene abundances than ML in the remaining seven pathways (*p* < 0.05). These findings demonstrate that high-rate mushroom residue combined with chemical fertilizer can more efficiently activate soil microbial carbon-metabolic functions.

The cbb3-type cytochrome c oxidase is mainly found in the phylum *Pseudomonadota*, covering typical genera such as *Bradyrhizobium* and *Pseudomonas*. As a high-affinity oxidase, it is related to bacterial adaptation to anaerobic or anoxic environments [41]. In this study, the gene abundance of cbb3-type cytochrome c oxidase in all five fertilization treatments was significantly higher than that in the original BS soil. This variation was highly consistent with the remarkable increase in the relative abundance of *Pseudomonas* under fertilization (Figure 1C). Different from the cbb3-type, the aa3-type cytochrome c oxidase has low oxygen affinity, is preferentially expressed under aerobic conditions, and possesses markedly higher energy-metabolic efficiency. Previous studies have demonstrated that *Pseudomonas aeruginosa* primarily depends on aa3-type cytochrome c oxidase for energy metabolism. This enzyme is up-regulated under oligotrophic conditions, facilitating bacterial growth and reproduction in nutrient-poor environments [42]. Our results revealed that sole chemical fertilizer application (GF) significantly decreased the gene abundance of aa3-type cytochrome c oxidase while increasing that of the cbb3-type compared with BS. This phenomenon can be explained by two aspects. First, long-term single chemical fertilization easily induces soil compaction and reduced porosity, deteriorating soil aeration capacity [43]. Second, urea hydrolysis releases gases such as CO_2_, which further displaces oxygen in soil pores and forms hypoxic microenvironments. Compared with GF, the combined application of mushroom residue and chemical fertilizer (MH, ML) significantly elevated the gene abundance of aa3-type cytochrome c oxidase. This suggests that mushroom residue amendment effectively improves topsoil aeration structure, increases soil oxygen concentration, optimizes aerobic soil conditions, and consequently regulates the expression patterns of microbial oxidase-related functional genes [4,44].

Our results showed that the gene abundance associated with the tricarboxylic acid (TCA) cycle in the original BS soil was significantly higher than that in all fertilization treatments (Figure 4L). The TCA cycle serves as the core energy-producing pathway for microbial aerobic metabolism and is closely linked to microbial tolerance to environmental stress [45]. Therefore, the high abundance of TCA-cycle-related genes in BS indicated stronger overall aerobic metabolic activity of soil microbes compared with fertilized soils, and implied that indigenous microorganisms might suffer greater environmental stress. Meanwhile, BS also maintained a relatively high abundance of genes involved in anaplerotic reactions. This suggested that TCA-cycle intermediates were largely diverted to biosynthetic pathways and continuously consumed. Microbes could activate anaplerotic pathways to timely replenish intermediates depleted during carbon metabolism, so as to sustain carbon metabolic homeostasis [46]. Combined with soil physicochemical analysis, the original soil was sampled from a greenhouse with a prior fertilization history. It possessed better nutrient status than ordinary loessial soil (Table 2) and favorable aeration conditions, which mainly explained the higher aerobic metabolic activity of indigenous microbes. The MH treatment exhibited significantly higher TCA-cycle-related gene abundance than other fertilization groups, with anaplerotic gene abundance comparable to BS. This indicated that high-rate mushroom residue combined with chemical fertilizer effectively alleviated hypoxic stress caused by sole chemical fertilization, improved the soil microenvironment, and restored the aerobic metabolic capacity of soil microorganisms.

Inter-group differences in gene abundance of the gluconeogenic pathway further verified the divergence in soil physicochemical properties among treatments. The BS group had significantly higher abundances of gluconeogenesis-related genes than all fertilization treatments, indicating a shortage of readily available carbon sources such as labile sugars in the original soil. Soil microorganisms thus synthesize glucose from non-carbohydrate substrates, including organic acids and lipids, through gluconeogenesis to maintain vital metabolic activities. Among all fertilized treatments, MH possessed significantly higher gluconeogenic gene abundance than other groups, demonstrating that high-rate mushroom residue amendment provides sufficient readily available carbon sources such as glucose and effectively optimizes soil nutrient composition. F-type ATPase couples ATP synthesis with electron transport and proton translocation in the bacterial respiratory chain. Elevated abundance of this enzyme strengthens microbial oxidative phosphorylation, compensates for insufficient energy production from substrate-level phosphorylation, and stabilizes microbial energy metabolism [47]. In this study, MH and ML showed significantly higher F-type ATPase gene abundances than other treatments. This was because the addition of mushroom residue markedly boosted soil microbial metabolic activity. Vigorous microbial growth and metabolism consume more energy, thereby inducing high expression of F-type ATPase-related genes to satisfy microbial energy requirements.

In summary, different fertilization regimes significantly regulate metabolic pathways and energy-metabolic traits of soil microorganisms. Overall, soil microbial metabolic patterns shifted toward hypoxic or anaerobic metabolism after fertilization, while microbes in the unfertilized original soil were mainly characterized by aerobic metabolism. Among all fertilization treatments, the combined application of high-rate mushroom residue and chemical fertilizer exhibited significantly higher levels of soil microbial aerobic metabolism than other fertilization modes. Based on the above results, we propose that proper soil tillage should be carried out after field fertilization to effectively enhance soil aeration, improve oxygen conditions, promote the transition of microbial metabolism from anaerobic to aerobic pathways, and optimize soil microbial metabolic functions. In addition, metagenomic sequencing only reflects the abundance profile of microbial functional genes and cannot directly characterize real-time microbial metabolic activity. Thus, soil microbial metabolic patterns can only be reasonably inferred from gene abundance data. Future studies could combine metatranscriptomic sequencing to further dissect microbial metabolic response mechanisms to different fertilization practices at the gene expression level, so as to more accurately and comprehensively uncover the metabolic regulation rules of soil microorganisms under varied fertilization treatments.

### 3.5. Correlation Between Soil Environmental Factors, Microbial Communities, and C-N-P-S Metabolism

**Figure 5:**
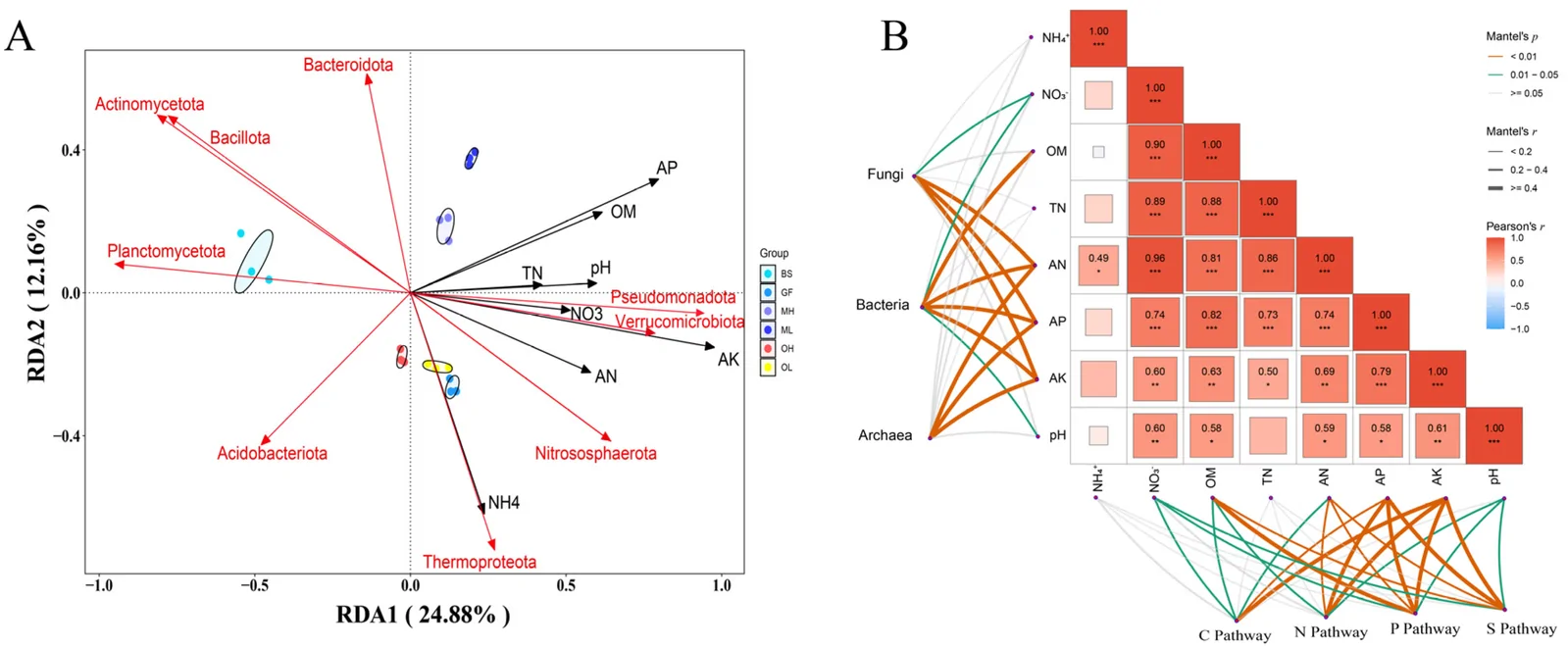
Correlation analysis between soil physicochemical factors, microbial communities, and carbon-nitrogen-phosphorus-sulfur metabolism. (**A**) RDA analysis; (**B**) Mantel test. AK, available potassium; AP, available phosphorus; ${\mathrm{NO}}_{3}^{-}$, soil nitrate nitrogen; AN, alkaline hydrolysis nitrogen; TN, total nitrogen; OM, organic matter; ${NH}_{4}^{+}$, ammonium nitrogen. *, **, and *** indicate significant differences at *p* < 0.05, *p* < 0.01 and *p* < 0.001, respectively.

Redundancy analysis (RDA) was performed to explore the relationships between soil environmental factors and microbial taxa in this study, and the detailed results are shown in Figure 5A. The first axis (RDA1) and the second axis (RDA2) explained 24.88% and 12.16% of the variation in soil physicochemical properties, respectively. AP, AK, and ${NH}_{4}^{+}$-N were the core environmental factors driving the differentiation of soil microbial communities under different treatments. Significant positive correlations existed among soil ${\mathrm{NO}}_{3}^{-}$-N, AN, TN, OM, and available AP, which was consistent with the findings of Ren et al. [44]. Correlation analysis between microbial communities and environmental factors showed that *Actinomycetota*, *Bacillota,* and *Planctomycetota* were negatively correlated with OM, AP, pH, and TN. These phyla were dominant taxa in the original BS soil, implying that they were more adapted to oligotrophic soil habitats with poor nutrient availability. *Pseudomonadota* and *Verrucomicrobiota* were positively correlated with OM, AP, pH, AK, and ${\mathrm{NO}}_{3}^{-}$-N. They were the dominant phyla in high-rate mushroom residue treatments (MH, ML) and tended to enrich in soils with high organic matter and nutrient levels. Some previous studies reported that the abundance of *Pseudomonadota* was negatively correlated with soil nutrient indicators such as total nitrogen, total phosphorus, total potassium, and organic matter [48], which was contrary to our results. This indicated that differences in soil types and fertilization regimes could significantly change the response relationships between microorganisms and environmental factors, even resulting in completely opposite outcomes. *Nitrososphaerota* and *Thermoproteota* exhibited significant positive correlations with ${NH}_{4}^{+}$-N and AN, yet negative correlations with OM and AP. They dominated treatments of sole chemical fertilizer, low-rate organic manure, and low-rate mushroom residue (GF, OH, OL), and were more suitable for soil environments with high ${NH}_{4}^{+}$-N and AN contents. *Nitrososphaerota* are key functional microorganisms in soil nitrogen cycling, which can transform ${NH}_{4}^{+}$-N into nitrite-nitrogen under anaerobic conditions [49]. Most archaea belonging to *Thermoproteota* are anaerobic heterotrophic microorganisms that utilize proteins and carbohydrates for growth, while a few taxa participate in chemolithoautotrophic processes of soil sulfur cycling [50]. Although ${NH}_{4}^{+}$-N acts as a substrate for *Nitrososphaerota*, several studies have demonstrated no significant correlation between ${NH}_{4}^{+}$-N content and the abundance of this phylum [51], which differed from our findings. We inferred that the significant positive correlations between ${NH}_{4}^{+}$-N and the two phyla in this study were mainly caused by decreased soil aeration after fertilization. The formed hypoxic and anaerobic microenvironments further remarkably promoted the enrichment and proliferation of these two anaerobic microbial groups.

To systematically reveal the intrinsic relationships among soil physicochemical factors, microbial community structure, and carbon-nitrogen-phosphorus-sulfur (C-N-P-S) metabolic pathways, the Mantel test was further conducted in this study (Figure 5B). Correlation analysis of environmental factors showed that soil ${NH}_{4}^{+}$-N was only significantly positively correlated with AN, with no significant correlations with other physicochemical factors. Except for ${NH}_{4}^{+}$-N and soil pH, all other soil physicochemical indicators presented significant positive pairwise correlations. Specifically, ${\mathrm{NO}}_{3}^{-}$-N, AN, TN, OM, and AP were extremely significantly positively correlated with one another (*p* < 0.001), and AP was also extremely significantly positively correlated with available potassium (AK) (*p* < 0.001). Soil pH was significantly positively correlated with all physicochemical factors except ${NH}_{4}^{+}$-N and TN (*p* < 0.05). Long-term field positioning experiments with mushroom residue-based organic manure have verified significant positive correlations among soil OM, AN, AP, and AK, which were consistent with our results. However, these nutrient indicators were significantly negatively correlated with pH in those studies, contrary to our findings [52]. Meanwhile, research on organic manure application has also confirmed significant correlations among soil organic carbon, AP, AK, and ${\mathrm{NO}}_{3}^{-}$-N [44]. Mechanistically, organic manure is rich in organic matter, nitrogen, phosphorus, potassium, and other nutrients [10]. Its input simultaneously increases multiple soil nutrient contents, resulting in universal positive correlations among nutrient indicators. Differences in initial soil background, fertilization regimes, and experimental duration were the primary reasons for the divergent responses of soil pH to nutrient elements. Mantel test between microbial communities and physicochemical factors revealed that AN, AP and AK had extremely significant strong correlations with bacterial, fungal and archaeal communities (Mantel’s *r* ≥ 0.4, *p* < 0.01). ${\mathrm{NO}}_{3}^{-}$-N was significantly correlated with bacterial and fungal communities, while pH was only significantly correlated with bacterial communities. Mantel test between microbial metabolic pathways and physicochemical factors indicated that ${NH}_{4}^{+}$-N and TN had no significant correlations with soil microbial C-N-P-S metabolic pathways. OM, AN, AP, and AK were key environmental factors regulating these pathways. Among them, OM mainly drove variations in phosphorus metabolism, AP significantly affected nitrogen, phosphorus, and sulfur metabolism, and AK showed strong correlations with all C-N-P-S metabolic pathways.

## 4. Conclusions

This study demonstrated that short-term different fertilization treatments significantly altered physicochemical properties, microbial community structure, and metabolic characteristics of greenhouse soils. Combined application of organic fertilizer with chemical fertilizer and mushroom residue with chemical fertilizer effectively increased soil organic matter as well as nitrogen, phosphorus and potassium contents, and optimized soil nutrient composition, whereas high-rate mushroom residue addition tended to induce soil alkalization. Fertilization regimes had crucial impacts on dominant soil microbial communities. Combined application of mushroom residue and chemical fertilizers enriched beneficial microbes, enhanced soil microbial diversity, and improved soil microecology. Fertilization remarkably reshaped microbial carbon-nitrogen-phosphorus-sulfur (C-N-P-S) metabolic functions. Sole chemical fertilizer application favored anaerobic metabolism, while mushroom residue amendment improved the soil aeration microenvironment and restored microbial aerobic metabolic levels. Soil nutrient indicators acted as core drivers of microbial community and functional differentiation. In summary, the combined application of mushroom residue and chemical fertilizer can ameliorate greenhouse soil quality. Nevertheless, mushroom residue dosage should be rationally controlled, and soil tillage is recommended to optimize soil oxygen conditions.

Although metagenomic sequencing can comprehensively characterize the profile of all functional genes in soil, it fails to capture the gene expression levels. Therefore, microbial functional analysis based solely on metagenomic results has certain limitations. Future studies should select specific functional genes for real-time quantitative PCR (qPCR) assays according to metagenomic data to determine the expression levels of key functional genes, or combine metatranscriptomic sequencing technology to quantify the expression of functional genes. This approach can yield more accurate insights into the variations of soil microbial metabolic functions.

## Figures and Tables

**Table 1:** Fertilizer application rates of different treatments (Unit: kg/hm^2^).

Treatment	Chemical Fertilizer			Mushroom Residue	Organic Fertilizer
	Urea	Calcium Superphosphate	Potassium Sulfate		
GF	180	90	120	0	0
MH	180	90	120	16,000	0
ML	180	90	120	7500	0
OH	180	90	120	0	15,000
OL	180	90	120	0	7500

**Table 2:** Soil physicochemical properties under different fertilization methods.

Treatments	AK (mg/kg)	AP (mg/kg)	${\mathrm{NO}}_{3}^{-}$-N (mg/kg)	AN (mg/kg)	TN (g/kg)	OM (g/kg)	${NH}_{4}^{+}$-N (mg/kg)	pH
BS	226.88 ± 4.42 e	123.46 ± 2.55 d	58.32 ± 1.91 e	83.94 ± 2.41 d	0.82 ± 0.05 d	10.77 ± 0.57 e	0.77 ± 0.02 c	8.07 ± 0.04 b
GF	372.93 ± 0.74 a	182.15 ± 4.03 b	96.09 ± 2.25 c	151.09 ± 3.58 b	1.1 ± 0.11 bc	11.68 ± 0.67 cd	14.68 ± 0.75 a	8.19 ± 0.04 ab
MH	355.99 ± 9.12 b	200.43 ± 2.61 a	127.93 ± 10.79 a	163.73 ± 10.99 a	1.35 ± 0.06 a	21.1 ± 1.12 a	0.86 ± 0.02 c	8.26 ± 0.06 a
ML	353.23 ± 1.63 b	198.64 ± 1.05 a	77.48 ± 1.2 d	109.47 ± 1.22 c	0.99 ± 0.13 cd	15.73 ± 0.79 bc	0.72 ± 0.02 c	8.18 ± 0.04 ab
OH	331.19 ± 1.68 d	178.94 ± 2.62 b	106.2 ± 3.29 b	145.6 ± 3.88 b	1.23 ± 0.12 ab	16.79 ± 0.57 b	5.53 ± 0.4 b	8.18 ± 0.11 ab
OL	344.78 ± 2.73 c	145.05 ± 2.05 c	73.94 ± 1.57 d	106.76 ± 1.77 c	0.86 ± 0.07 d	13.73 ± 0.7 d	0.68 ± 0.06 c	8.18 ± 0.04 ab

Values are presented as the mean ± standard deviation (*n* = 3). Different lowercase letters within a column for the same factor indicate significant differences (*p* < 0.05) based on a one-way ANOVA, followed by an LSD test. AK, available potassium; AP, available phosphorus; ${\mathrm{NO}}_{3}^{-}$-N, soil nitrate nitrogen; AN, alkaline hydrolysis nitrogen; TN, total nitrogen; OM, organic matter; ${NH}_{4}^{+}$-N, ammonium nitrogen. BS, original soil; GF, conventional chemical fertilizer; MH, high-rate mushroom residue combined with chemical fertilizer; ML, low-rate mushroom residue combined with chemical fertilizer; OH, high-rate organic manure combined with chemical fertilizer; OL, low-rate organic manure combined with chemical fertilizer.

## Data Availability

The raw sequence data reported in this paper have been deposited in the Genome Sequence Archive in the National Genomics Data Center, China National Center for Bioinformation/Belling Institute of Genomics, Chinese Academy of Sciences (GSA: CRA CRA042386), which are publicly accessible at https://ngdc.cncb.ac.cn/gsa, accessed on 7 May 2026.
